# PCSK9 inhibition as a novel therapeutic target for alcoholic liver disease

**DOI:** 10.1038/s41598-019-53603-6

**Published:** 2019-11-20

**Authors:** Ji Soo Lee, Partha Mukhopadhyay, Csaba Matyas, Eszter Trojnar, Janos Paloczi, Yuan Ru Yang, Brandon A. Blank, Cody Savage, Alexander V. Sorokin, Nehal N. Mehta, Janaina C. M. Vendruscolo, George F. Koob, Leandro F. Vendruscolo, Pal Pacher, Falk W. Lohoff

**Affiliations:** 1 Section on Clinical Genomics and Experimental Therapeutics, National Institute on Alcohol Abuse and Alcoholism, National Institutes of Health, Bethesda, MD USA; 2 Laboratory of Cardiovascular Physiology and Tissue Injury, National Institute on Alcohol Abuse and Alcoholism, National Institutes of Health, Bethesda, MD USA; 3 Neurobiology of Addiction Section, National Institute on Drug Abuse, National Institutes of Health, Baltimore, MD USA; 4 Lipoprotein Metabolism Section, National Heart, Lung and Blood Institute, National Institutes of Health, Bethesda, MD USA; 5 Section of Inflammation and Cardiometabolic Diseases, National Heart, Lung and Blood Institute, National Institutes of Health, Bethesda, MD USA

**Keywords:** Alcoholic liver disease, Addiction

## Abstract

Alcoholic liver disease (ALD) causes significant morbidity and mortality, and pharmacological treatment options are limited. In this study, we evaluated the PCSK9 inhibitor alirocumab, a monoclonal antibody that robustly reduces low-density lipoprotein cholesterol (LDL-C), for the treatment of ALD using a rat model of chronic alcohol exposure. Alirocumab (50 mg/kg) or vehicle was administered weekly for 6 weeks to rats receiving a 12% alcohol liquid diet or an isocaloric control diet. At the end of the alcohol exposure protocol, serum and liver samples were obtained for molecular characterization and histopathological analysis. PCSK9 inhibition with alirocumab attenuated alcohol-induced hepatic triglyceride accumulation through regulation of lipid metabolism (mRNA expression of modulators of fatty acid synthesis (FAS) and catabolism (PPARα and CPT1)), hepatocellular injury (ALT), hepatic inflammation (mRNA expression of pro-inflammatory cytokines/chemokines (TNFa, IL-1β, IL-22, IL-33, IL-17α, IL-2, MIP-2, and MCP-1), and neutrophil infiltration (myeloperoxidase staining)). Alirocumab treatment also attenuated alcohol-induced PCSK9 mRNA elevation and upregulated LDL-receptor (LDL-R) via modulation of the transcription factors (SREBP-1, SREBP-2, and E2F1) in liver. We demonstrated that chronic anti-PCSK9 treatment using the monoclonal antibody alirocumab attenuated alcohol-induced steatohepatitis in the rat model. Given the large unmet clinical need for effective and novel treatments for ALD, anti-PCSK9 treatment with the monoclonal antibody that spares liver metabolism is a viable new therapeutic possibility. Future studies are needed to elucidate the exact role of PCSK9 in ALD and alcohol use disorder (AUD) and to evaluate efficacy and safety of anti-PCSK9 treatment in clinical populations with ALD/AUD.

## Introduction

Alcoholic liver disease (ALD) causes significant morbidity and mortality and is the leading cause of cirrhosis, liver cancer, and acute/chronic liver failure^1–5^. Although the pathophysiology of ALD is clearly linked to excessive alcohol consumption, the exact mechanisms remain elusive and span domains of behavior as well as environmental, genetic, and epigenetic factors^6,7^. Treatment options for ALD are limited and mainly include abstinence from alcohol – a goal that is difficult to achieve for most individuals with ALD and/or alcohol use disorder (AUD). There are only limited pharmacological treatment options available for ALD, which, depending on the degree of liver damage, include corticosteroids, pentoxifylline, and N-acetylcysteine^8–13^. Once liver cirrhosis is present, liver transplant is often the only option. Given the large unmet clinical need for effective new pharmacological interventions for ALD, innovative approaches to identify novel targets and treatments are needed^14^.

We recently conducted an epigenome-wide association study of individuals with AUD and identified proprotein convertase subtilisin/kexin 9 (PCSK9) as a main target that is epigenetically regulated by alcohol consumption^15^. PCSK9 is primarily expressed in the liver^16–18^ and plays an integral part in low-density lipoprotein cholesterol (LDL-C) regulation by targeting the LDL-receptor (LDL-R) and subsequently modulating LDL-R expression and recycling^18–21^. Its role in lipid regulation was initially discovered by gain-of-function mutations in *PCSK9* that cause autosomal dominant hypercholesterolemia^22^ and loss-of-function mutations that are associated with a decrease in LDL-C levels and low rates of cardiovascular disease^23,24^. These findings have led to the rapid development of anti-PCSK9 therapeutics, which resulted in new ways of powerfully lowering LDL-C^25–29^.

Given alcohol’s direct toxic effects on liver tissue and often observed downstream effects of chronic alcohol consumption on lipid metabolisms, such as steatohepatitis, fatty liver disease and/or abnormal lipoprotein function^1^, PCSK9 is a plausible new target to investigate in ALD/AUD. In this study, we tested the hypothesis that anti-PCSK9 treatment (alirocumab) would have effects on liver endpoints relevant to ALD using a rat model of ALD/AUD.

## Materials and Methods

### Animals

Adult Sprague Dawley male rats at the age of 2 months obtained from Charles River (Kingston, NY) were used for the experiment. Before the beginning of the experiment, the rats were maintained in a room with a 12 h/12 h light/dark cycle (lights on at 7:00). The animals had continuous access to food and water prior to the beginning of the experiment. All animal experiments were approved by the National Institute on Alcohol Abuse and Alcoholism Animal Care and Use Committee. All procedures were performed according to the guidelines of the National Institutes of Health (NIH) Guide for the Care and Use of Laboratory Animals.

### Alcohol liquid diet and drug treatment

The rats received water and a nutritionally balanced liquid diet as the sole source of calories in their home cages (*ad libitum*). The alcohol liquid diet contained 5.25 g of salt (NaCl), 3.25 g of vitamins (MP biomedical LLC, Solon, OH), 15.17 g of sweetener, 302.25 ml of water, 52.25 ml of 95% v/v ethanol for 5% v/v and 126.31 ml for 12% v/v, and 592.50 ml of boost chocolate (BOOST® ORIGINAL, Nestle Health Science, MI) per liter. The control liquid diet contained the same ingredients except for 15.17 g of sweetener, and 121 g of sucrose was used instead of 95% v/v ethanol for an isocaloric match to the alcohol liquid diet. The ingredients were mixed well, kept refrigerated, and used for up to 3 days. The consumption of the liquid diet was measured and given fresh daily. The amount of liquid diet for the control rats was limited to match the same amount of calories consumed by the alcohol-exposed rats. For the alcohol groups, the rats received 5% of ethanol during a 5-day acclimation period. A liquid diet that contained 12% of ethanol was thereafter given for 6 weeks. Rodent chow was removed from the cages for the 5-day alcohol liquid diet exposure. During the weekends, regular chow was given back for 2 days to maintain healthy body-weight. Alirocumab (Praluent®) was purchased from Regeneron Pharmaceuticals (Tarrytown, NY, USA) through the Division of Veterinary Resources at the NIH. Control and alcohol-treated rats were subcutaneously injected with alirocumab (50 mg/kg) or saline (used as vehicle) weekly prior to exposure to the liquid diets including the acclimation period at 5% of ethanol. The volume of the injections was 0.33 ml/kg for Praluent® 150 mg/ml solution.

### Blood sample collection

To determine blood alcohol concentrations, non-anesthetized rats were gently held and a razor blade was used to nick the tip of the tail (1 mm from the tip). Once the nick was made, the tail was gently stroked from the base toward the tip with fingers on each side of the tail to increase blood flow from the lateral vein. A volume of up to 300 µl/day (for a 300 g rat) of blood was collected. Hemostasis was achieved by gently drying the tail tip with paper towel or Kim wipe.

### Measurement of blood alcohol levels

Blood was collected in tubes and immediately put on ice and centrifuged for 10–15 mins. Following calibration with a standard, the separated plasma sample was injected into an oxygen-rate alcohol analyzer (Analox Instruments) to determine blood alcohol levels (BALs).

### Determination of liver injury

In a non-survival procedure, blood was collected from the inferior vena cava and serum was prepared for further analysis. Serum alanine aminotransferase (ALT) and aspartate aminotransferase (AST) levels were measured by using the Idexx VetTest 8008 (Idexx Laboratories, Westbrook, ME, USA) chemistry analyzer.

### Assesment of non-fasting serum lipid levels

Total cholesterol, free cholesterol, triglyceride, and non-esterified fatty acids concentrations from serum were determined by enzymic methods using commercial kits (FUJIFILM Wako Pure Chemical Corporation, Osaka, Japan) according to manufacturer’s instructions. Cholesteryl ester values were calculated by subtracting free cholesterol from total cholesterol concentrations. Serum lipid profile is shown in Table 1.

**Table 1:** Lipid Profiles of the 12% alcohol liquid diet rat model (non-fasting).

Variables	Con	Con + Drug	ETOH	ETOH + Drug	*P*-value
**Serum**					
Total Cholesterol (mg/dL)	52.43 ± 13.8	52.84 ± 19.17	54.39 ± 12.21	42.22 ± 17.73	0.4209
Free Cholesterol (mg/dL)	0.08 ± 0.13	0.08 ± 0.11	0.09 ± 0.17	0.05 ± 0.08	0.9960
Cholesteryl Esters (mg/dL)	52.35 ± 13.69	52.76 ± 19.10	54.30 ± 12.12	42.18 ± 17.73	0.4204
Triglyceride (mg/dL)	63.13 ± 22.49	35.20 ± 19.26	103.80 ± 27.64^#^	181.1 ± 80.14^###^	0.0003
Non-esterified fatty acid (mEq/L)	0.38 ± 0.10	0.54 ± 0.16	0.31 ± 0.08^##^	0.36 ± 0.11^#^	0.0031
**Liver**					
Total Cholesterol (ug/mg tissue)	21.86 ± 13.33	40.63 ± 16.59	45.74 ± 16.93*	44.05 ± 19.15	0.0287
Free Cholesterol (ug/mg tissue)	17.90 ± 11.41	37.18 ± 16.05	37.30 ± 13.40	37.95 ± 17.32	0.0276
Cholesteryl Esters (ug/mg tissue)	3.96 ± 2.73	3.45 ± 1.73	8.44 ± 4.51*	6.10 ± 3.47	0.0198
Triglyceride (nmol/g tissue)	2.18 ± 0.46	1.95 ± 0.56	5.03 ± 1.01***	3.45 ± 0.45**	< 0.0001

Serum and liver tissues were used to measure various parameters. Rats were fed overnight before sacrificed for sample collection. Data are presented as mean ± S.D. Kruskal-Wallis test followed by Dunn’s multiple comparisons or ANOVA followed by Tukey’s posthoc test was applied to compare pairs of the group mean. In the table column, overall *P*-values are stated. *P < 0.05 compared to Con group, ***P < 0.001 compared to Con group, **P < 0.01 compared to ETOH group, ^#^P < 0.05 compared to Con + Drug group, ^##^P < 0.01 compared to Con + Drug group, and ^###^P < 0.001 compared to Con + Drug group.

### Determination of hepatic lipid levels

Triglyceride content of liver sample homogenates was determined using a commercially available kit (#K622-100, Biovision, San Francisco, CA, USA) according to the manufacturer’s protocol^30^. Liver cholesterol levels were measured using Cholesterol/Cholesteryl Ester Quantification Kit (#ab65359, Abcam Inc., Toronto, Ontario, Canada). Briefly, 30–40 mg liver tissues were extracted in 600–800 ul Chloroform: Isopropanol: NP-40 (7:11:0.1) using a micro homogenizer. 400 ul extracted tissues were transferred to a 1.5 ml tube and spun at 15,000 g for 5 mins. All of the liquid (organic phase) was transferred to a new 1.5 ml tube and dried at 50 °C in a nitrogen evaporator to remove chloroform. Dried extract was dissolved in 400 µl assay buffer by vortexing. This sample solution was diluted 20 times using the assay buffer and assayed as described by the manufacture. Cholesteryl ester values were calculated by subtracting free cholesterol from total cholesterol concentrations. Hepatic lipid profile is presented in Table 1.

### RNA isolation and cDNA

Rat tissues were homogenized in TRI Reagent® (Zymo Research Corp) with ceramic beads using Precellys 24 Homogenizer at 4–8 °C. RNAs were isolated using Direct-zol™ RNA MiniPrep kit according to manufacturer’s instruction including in-column DNase treatment (Zymo Research Corp). cDNAs were prepared using High-Capacity cDNA Reverse Transcription Kit (Thermo Fisher Scientific) from those isolated RNAs.

### Real-time PCR

Real-time PCRs of target genes were performed by SYBER green method using primers and SYBER green PCR master mix (Thermo Fisher Scientific). The experiments were performed in duplicate for each sample using the ABI PRISM 7900 Real-Time PCR System as described earlier^30^. The specificity of transcript amplification was confirmed by dissociation curve profiles. The expression levels of target genes were normalized to the average expression of four housekeeping genes (beta-actin (Actb), 60 S acidic ribosomal protein large P1 (Rplp), beta-2-microtubulin (B2m), and glyceraldehyde-3-phosphate dehydrogenase (GAPDH)) and calculated based on the comparative cycle threshold Ct method (2−ΔΔCt). Primer details are provided in Table 2.

**Table 2:** qRT-PCR primers used in the paper.

Genes Rat	Primer info	
IL1b	Catalog # QT00181657	Qiagen
TNFa	Catalog # QT02488178	Qiagen
IL17a	Catalog # QT02544185	Qiagen
IL22	Catalog # QT01597071	Qiagen
IL10	Catalog# QT00177618	Qiagen
Actb	Catalog# QT00193473	Qiagen
Rplp	Catalog# QT01745625	Qiagen
B2m	Catalog# QT00176295	Qiagen
	**Forward primer (5′-3′)**	**Reverse primer (5′-3′)**
GAPDH	ACAAGATGGTGAAGGTCGGTG	TCCCATTCTCAGCCTTGACTG
IL33	GTGGATGGGAAGAAGCTGATG	GCATTCAGCCAGATGTCTGTGT
MCP1	TCTCTCTTCCTCCACCACTATGC	GGCAGCAACTGTGAACAACAG
MIP2	TGAACAAAGGCAAGGCTAACTG	GATTCTGCCCGTTGAGGTACAG
IL2	CTGACGCTTGTCCTCCTTGTC	GGTGCTGCTGTGTTTCCTTTG
PCSK9	CATGGAACCTGGAGCGGATT	ACCTGGCTACTTCCGTCAGG
LDLR	CGCAGCCTAGAGGGGTAAAC	GAGTGGGCACTGATCTGAGG
SREBP-2	GTGACTGAGAGTCCCTTGGTG	AGGAGTTCTGTTGCCCATCG
FAS	GCCTAACACCTCTGTGCAGT	GGCAATACCCGTTCCCTGAA
PPARα	CTGTCCGCTACTTCGAGTCC	GAACCCTCCAGCCCACAAAA
CPT1	GCAGAGCAATAGGTCCCCAC	CATCGGGGGTGACAGTGAAC
CD36	ACTGTGGCTAAATGAGACTGGG	CCCGGTCACTTGGTTTCTGA
ACT2	GACTACTGCTGAGCGTGAGA	AGAAGAGGAAGCAGCAGTGG
COL1a1	CATGGCCAAGAAGACATCCC	TCAGGTTTCCACGTCTCACC
MMP2	TGGCACCACCGAGGATTATG	CCCACAGTGGACATAGCAGT
TIMP	CGCAGCGAGGAGTTTCTCAT	AGCAGGGCTCAGATTATGCC
SREBP-1	ATTTGGCCCGGGGAGATTTT	CAGGCCAGATCCAGGTTTGA
E2F1	GTGAAACGGAGGCTGGATCT	TTTCACACCTTTCCCTGGGT

### Western blot

Liver samples were homogenized in RIPA buffer containing Complete Protease and PhosStop Phosphatase Inhibitor Cocktail (Roche). Protein concentration was determined using Pierce BCA Protein Assay Kit (Thermo Fisher Scientific). Samples (20 μg protein) in 4x Laemmli SDS Sample Buffer (Bio-Rad) were loaded on Criterion™ TGX™ Tris-Glycine 4–20% precast gels (Bio-Rad). Proteins were separated in SDS-PAGE and transferred to nitrocellulose membrane (Trans-Blot® Turbo™ Transfer System, Bio-Rad). Membrane was blocked (SuperBlock TBS Blocking buffer, Thermo Fisher Scientific) for 30 mins at room temperature and incubated with primary antibodies (anti-LDLR: Abcam, #ab180623, 1:2000; anti-GAPDH: Millipore, MAB374, 1:10000) in the blocking buffer overnight at 4 °C with gentle agitation. Next day, the membrane was incubated with secondary antibodies (anti-rabbit IgG: 1:2000, #7074; anti-mouse IgG – 1:10000, #7076, Cell Signaling Technology) for 1 hour at room temperature in TBS buffer containing 2.5% milk and 0.1% Tween 20. Signal was developed by enhanced chemiluminescence detection method (Supersignal West Dura, Thermo Fisher Scientific) and captured with G:BOX mini imaging system (Syngene, Frederick, MD, USA). Band densities were analyzed by GeneTools software (Syngene), and GAPDH was used as a loading control.

### Hepatic 4-hydroxynonenal (HNE) content

Levels of hepatic 4-HNE were measured by using the kit from Cell Biolabs, San Diego, CA, USA as described earlier^31^. The results were expressed as fold change compared to the control group.

### Liver histology and immunohistochemistry

Rat liver samples were fixed in 10% neutral buffered formalin. After embedding and cutting 5 µm slices, all sections were deparaffinized and stained with the conventional haematoxylin/eosin staining method. For myeloperoxidase (MPO) immunostaining, sections were deparaffinized, and re-hydrated in descending grades of ethanol, followed by heat mediated antigen retrieval procedure. To block endogenous peroxidase activity, sections were incubated in BloxALL solution (Vector Laboratories, Burlingame, CA, USA) according to the manufacturer’s instruction. Sections were then incubated with anti-MPO antibody (1:500 dilution; ab 188211, Abcam, Cambridge, MA, USA); or anti-4-HNE antibody (1:100 dilution, MHN-100P, Genox corporation) overnight at 4 °C in a humidified chamber. Sections were incubated with an anti-rabbit or anti-mouse IgG conjugated with a horseradish-peroxidase polymer (ImmPress reagents, Vector Laboratories) according to the kit’s instructions. Color development was induced by incubation with a DAB reagent (Vector Laboratories) for 30–60 seconds, and the sections were counter-stained with haematoxylin. Finally, the sections were dehydrated in ethanol, cleared in xylene, and mounted. For Sirius Red statining, rat liver sections were stained with Sirius Red in order to visualize hepatic fibrosis. Briefly, the sections were stained in picro-sirius red solution for 2 hours. After the washing steps with 10% glacial acetic acid, the sections were dehydrated through 3 changes of 100% ethanol and xylene then mounted in a resinous medium. All images were captured by using an Olympus BX-43 microscope set (Olympus, Center Valley, PA), and 7–10 random areas were taken. Both MPO positive cells and sirius red coverage were quantified by ImageJ software (NIH, Bethesda, MD, USA).

### Statistical analysis

All data were expressed as mean ± S.E.M. Sample number represents the number of animals. Kruskal-Wallis test followed by Dunn’s multiple comparisons was applied for non-normally distributed data. One-way analysis of variance (ANOVA) was used for multiple group comparisons with Tukey’s posthoc test when data were normally distributed. The analyses were performed using GraphPad-Prism 7.0 (GraphPad Software, Inc., San Diego, CA). Significant differences were determined statistically at p < 0.05.

## Results

### Experimental paradigm and baseline characteristics of animals

**Figure 1:**
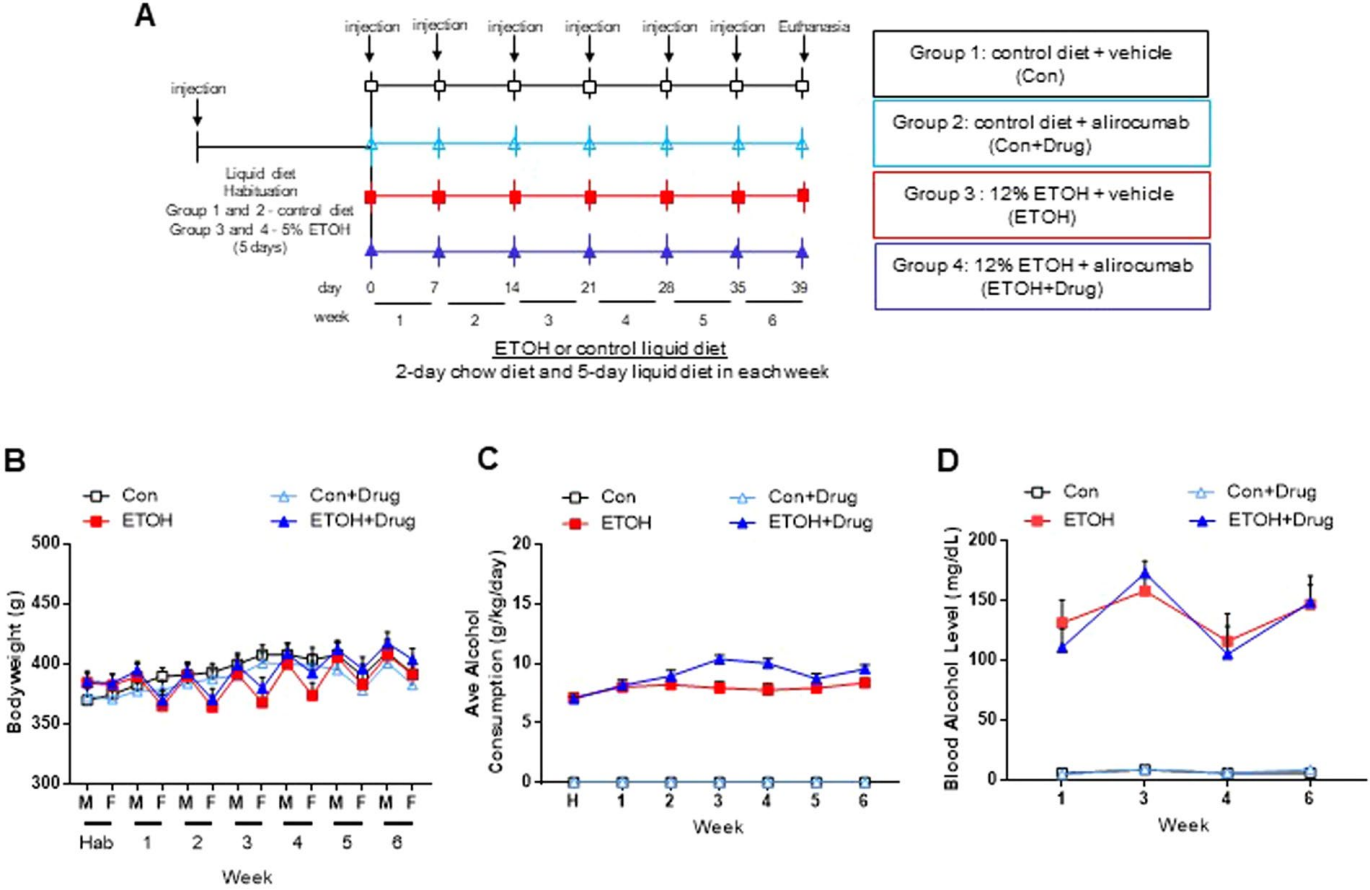
Experimental paradigm and baseline characteristics of rats. (**A**) Schematic showing the experimental design. Vehicle or PCSK9 inhibitor (alirocumab, 50 mg/kg) was subcutaneously injected to rats every week prior to chronic alcohol exposure. The rats were given 5% of alcohol for 5 days during the habituation period and then 12% alcohol for 6 weeks. **(B)** Body weights measured twice a week. **(C)** Average of daily alcohol consumption. **(D)** Blood alcohol level. *n* = 8 for all groups.

Rats were subcutaneously administered 50 mg/kg of PCSK9 inhibitor alirocumab or vehicle weekly at the beginning of alcohol exposure (Fig. 1A). The injection dose (50 mg/kg) was determined based on a previous study that reported no toxicity as well as efficacy in reducing cholesterol levels in Sprague Dawley rats^32^. Alcohol-fed rats initially received 5% of alcohol, which was lower than concentrations commonly used^33,34^ to habituate them to the taste of alcohol for 5 days, followed by 12% alcohol for 6 weeks; control groups remained in the control diet without alcohol. They were given the liquid diet for 5 days a week and the regular rat chow for 2 days to maintain healthy body-weight. (N = 8 for all groups; Fig. 1A). The detailed information is described in the methods. Body weights were measured twice a week and showed no statistically significant differences in all groups. Furthermore, rats lost weight during the liquid diet but gained during the chow diet (Fig. 1B). The alcohol treated groups drank the liquid diets between 6 and 10 g/kg per day (Fig. 1C), and their blood alcohol levels ranged from 100 to 170 mg/dl (Fig. 1D).

### Effect of PCSK9 inhibitor on hepatic mRNA/or protein expression of PCSK9, LDL-R, and transcription factors (SREBP-1, SREBP-2, and E2F1) in the chronic alcohol liquid diet model

**Figure 2:**
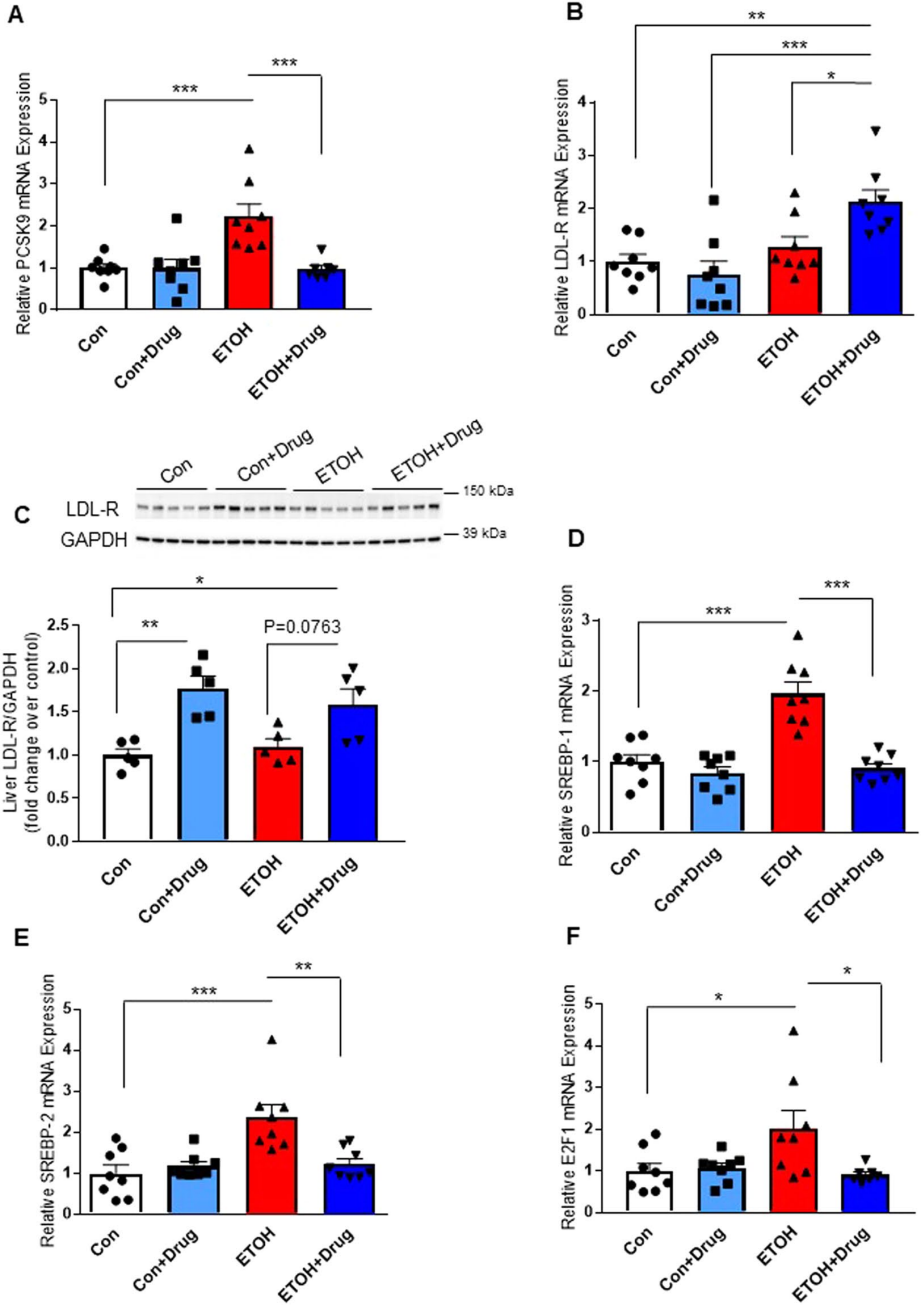
Effect of PCSK9 inhibitor on expression of PCSK9 and LDL-R in the liver. (**A**) Hepatic PCSK9 mRNA expression (n = 7 for Ethanol(ETOH) + Drug and 8 for the other groups). **(B)** Hepatic LDL-R mRNA expression (n = 8 for all groups). **(C)** Uncropped immunoblot images and analysis of hepatic LDL-R protein expression (n = 5 for all groups). Full-length blots are presented in Supplementary Figure. **(D)** Hepatic SREBP-1 mRNA expression (n = 8 for all groups). **(E)** Hepatic SREBP-2 mRNA expression (n = 8 for all groups). **(F)** Hepatic E2F1 mRNA expression (n = 7 for ETOH + Drug and 8 for the other groups). Data are means ± S.E.M. *P < 0.05, **P < 0.01, and ***P < 0.001. Statistical significance was determined by one-way ANOVA followed by Tukey’s posthoc comparisons.

Previously, we observed an increase in hepatic transcriptional expression of PCSK9 in rats chronically exposed to alcohol vapor^15^. Remarkably, hepatic PCSK9 mRNA expression also increased in rats fed 12% alcohol, which was attenuated by alirocumab treatment (Fig. 2A). Although the alcohol diet had no significant effect on hepatic LDL-R expression, treatment of alcohol exposed rats with alirocumab significantly increased hepatic mRNA and protein expression of LDL-R, consistent with alirocumab’s primary mechanism of action (Fig. 2B,C). Furthermore, to identify the underlying mechanism of regulation of PCSK9 and LDL-R expression, we measured gene expression of transcription factors (sterol regulatory element-binding protein-1 (SREBP-1), SREBP-2, and E2F transcription factor 1 (E2F1)) controlling cholesterol homeostasis. Hepatic mRNA expressions of SREBP-1, SREBP-2, and E2F1 were elevated in alcohol-fed rats, but alirocumab administration attenuated the alcohol-induced expressions of the transcription factors, which is consistent with previous studies^34–36^ (Fig. 2D–F).

### PCSK9 inhibition attenuates alcohol-induced lipid accumulation and regulates lipid metabolism

**Figure 3:**
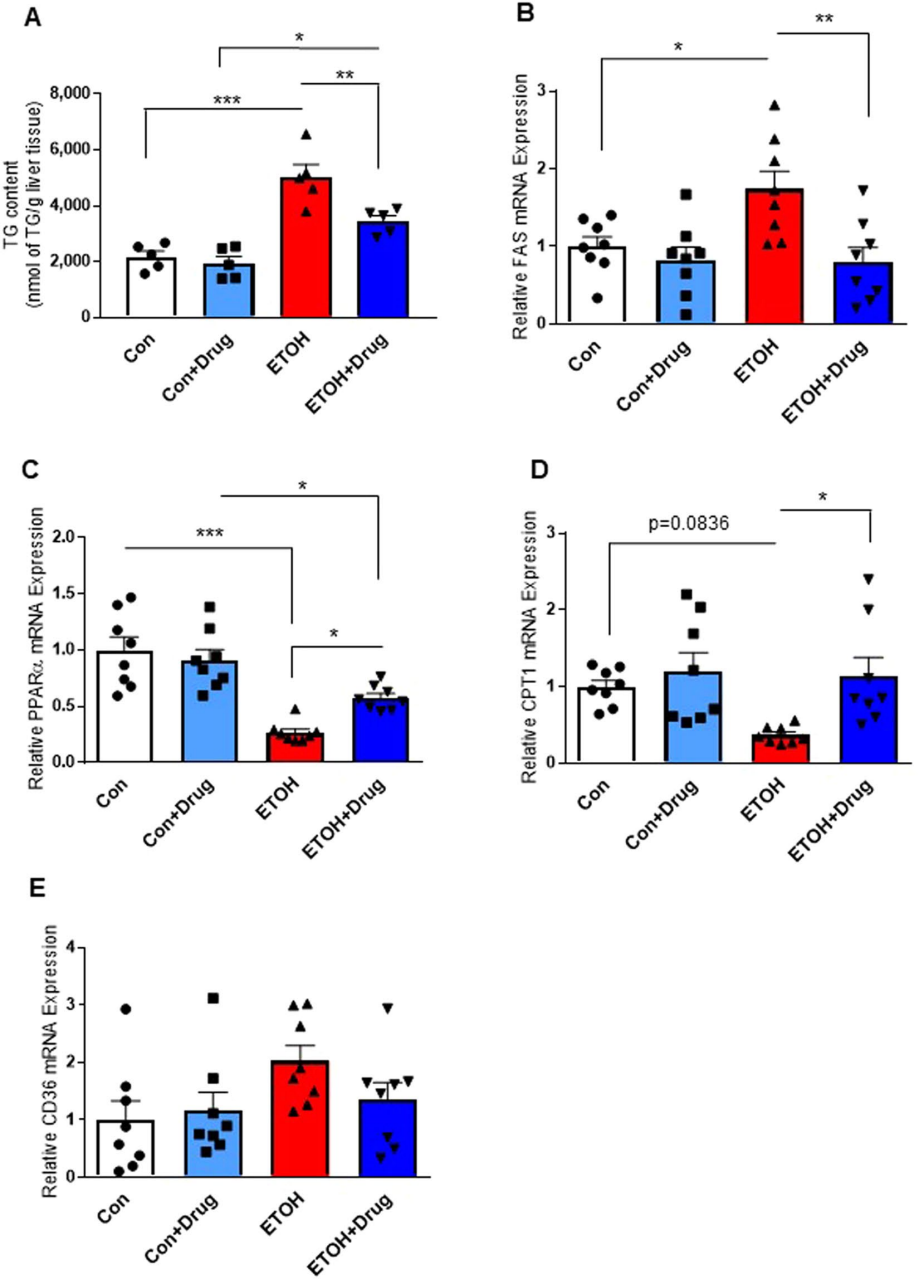
Effect of PCSK9 inhibitor on alcohol-induced fatty acid accumulation and metabolism. (**A**) Liver TG content in indicated groups (*n* = 5 for all groups) **(B–E)** Hepatic mRNA levels of FAS **(B)**, PPARα **(C)**, CPT1 **(D)**, CD36 **(E)** (n = 8 for all groups). Data are means ± S.E.M. *P < 0.05, **P < 0.01, and ***P < 0.001. Statistical significance was determined by one-way ANOVA with Tukey’s posthoc test.

Because PCSK9 was positively associated with plasma triglyceride (TG) levels^37–41^, we investigated whether the PCSK9 inhibitor would affect hepatic lipid contents in alcohol-fed rats. Alcohol exposure increased hepatic TG level, which was attenuated by alirocumab treatment (Fig. 3A and Table 1). We furthere examined an impact of the PCSK9 inhibitor on lipid metabolism. Gene expression of fatty acid synthase (FAS), a lipogenic enzyme important in TG accumulation, was increased in the alcohol fed-rat group, which was reduced by alirocumab administration (Fig. 3B). Moreover, alcohol exposure down-regulated mRNA expression of peroxisome proliferator-activated receptor alpha (PPARα) and carnitine palmitoyltransferase I (CPT1), regulators of fatty acid catabolism, wherease alirocumab treatment restored the reduction (Fig. 3C,D). However, there were no statistically significant differences in cluster of differentiation 36 (CD36) involved in fatty acid uptake (Fig. 3E). However, serum cholesterol levels of the drug treated groups were not statistically different from those of the vehicle treated groups (Table 1), which could be attributed to absence of overnight fasting before sample collection. Detailed serum and hepatic lipid profiles of the liquid diet model are presented in Table 1.

### PCSK9 inhibition attenuates alcohol-induced liver injury

**Figure 4:**
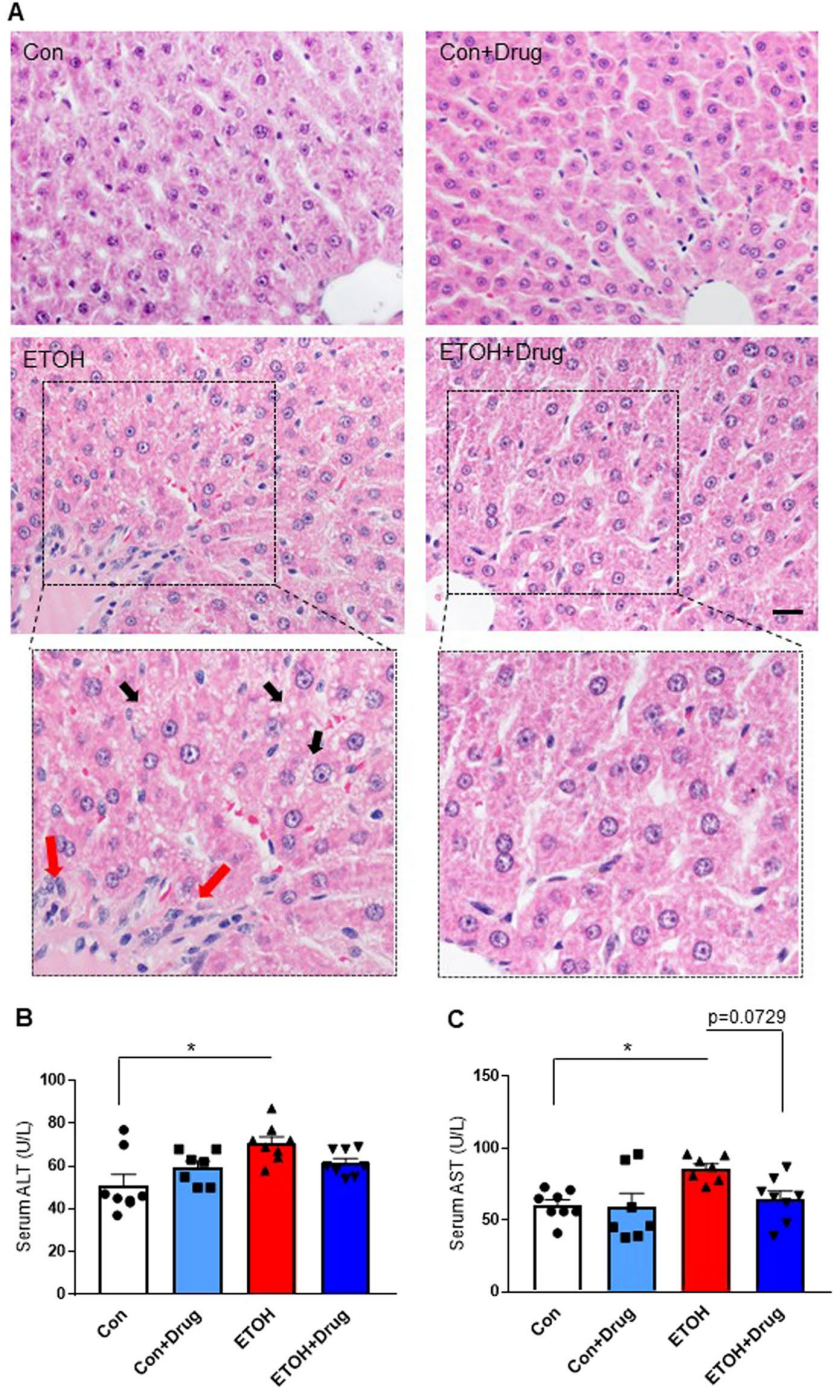
Effect of PCSK9 inhibitor on alcohol-induced liver injury. (**A**) Representative images of H&E stained liver sections from indicated groups. Scale, 20 µm. Enlarged view of boxed region is shown below. **(B)** Serum ALT levels in indicated groups (*n* = 7 for Control(Con) + Drug and 8 for the other groups). *P < 0.05. Statistical significance was determined by Kruskal-Wallis test followed by Dunn’s posthoc test. **(C)** Serum AST levels in indicated groups (*n* = 7 for Con + Drug and ETOH and 8 for Con and ETOH + Drug). *P < 0.05. Statistical significance was determined by one-way ANOVA with Tukey’s posthoc test. Data are means ± S.E.M.

To determine the efficacy of alirocumab on altering alcohol-induced hepatocellular injury and related endpoints, we first studied histological changes in liver tissues prepared with hematoxylin and eosin (H&E) staining^42,43^. As expected, rats chronically fed alcohol showed signs of hepatic steatosis (multiple small vacuoles indicated by the black arrows in the wide-field image) and inflammation (infiltrating leukocytes indicated by the red arrows in the wide-field image), which were attenuated by alirocumab treatment (Fig. 4A). Next, we confirmed the histological finding by using biomarkers for alcohol liver injury: serum ALT and AST^44^. Both serum ALT and AST levels were higher in the alcohol-fed group compared to the control group and were attenuated by alirocumab treatment (Fig. 4B,C). Alirocumab treatment had no effect in control animals on markers of liver injury (Fig. 4A–C).

### Assessment of effects on liver fibrosis

**Figure 5:**
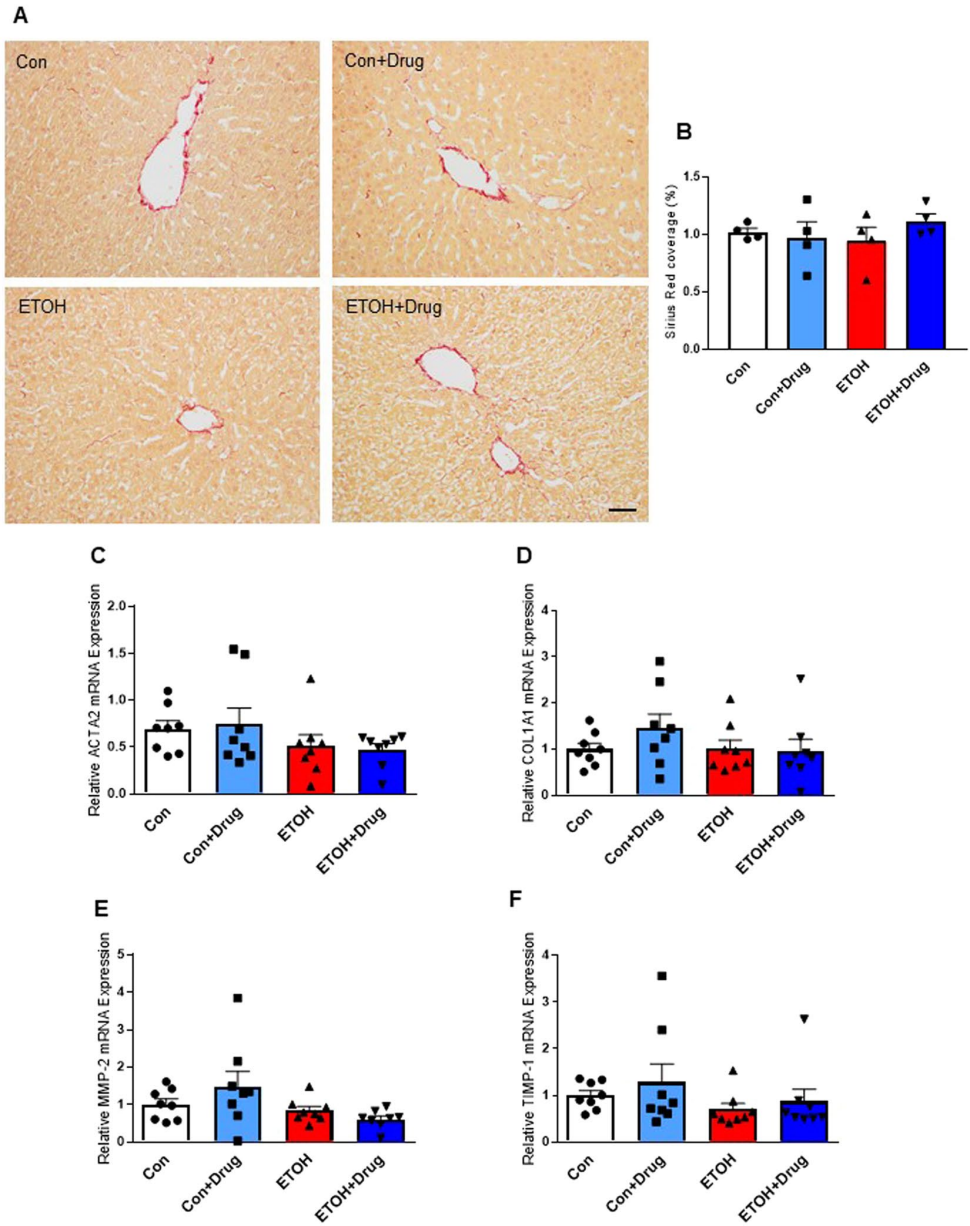
Effect of PCSK9 inhibitor on alcohol-induced liver fibrosis. (**A**) Representative images of rat liver tissues stained with Sirius Red from indicated groups. Scale bar, 50 µm. **(B)** Quantification of Sirius Red covered areas by histomorphometry (n = 4 for all groups). **(C–F)** Hepatic mRNA levels of ACTA2 **(C)**, COL1A1 **(D)**, MMP-2 **(E)**, TIMP-1 **(F)** (n = 8 for all groups). Data are means ± S.E.M.

We further examined the effect of PCSK9 inhibitor on alcohol-induced liver injuries by assessing liver fibrosis. We performed Sirius red staining to observe a degree of hepatic fibrosis determined by collagen deposition. None of the groups showed differences in collagen deposition quantified by Sirius red coverage (Fig. 5A,B). Consistent with the hitological outcome, there were no changes in gene expression of fibrosis markers, smooth muscle alpha-actin (ACTA2), alpha-1 type I collagen (COL1A1), matrix metalloproteinase-2 (MMP-2), and metallopeptidase inhibitor 1 (TIMP-1) (Fig. 5C–F).

### PCSK9 inhibition attenuates oxidative stress

**Figure 6:**
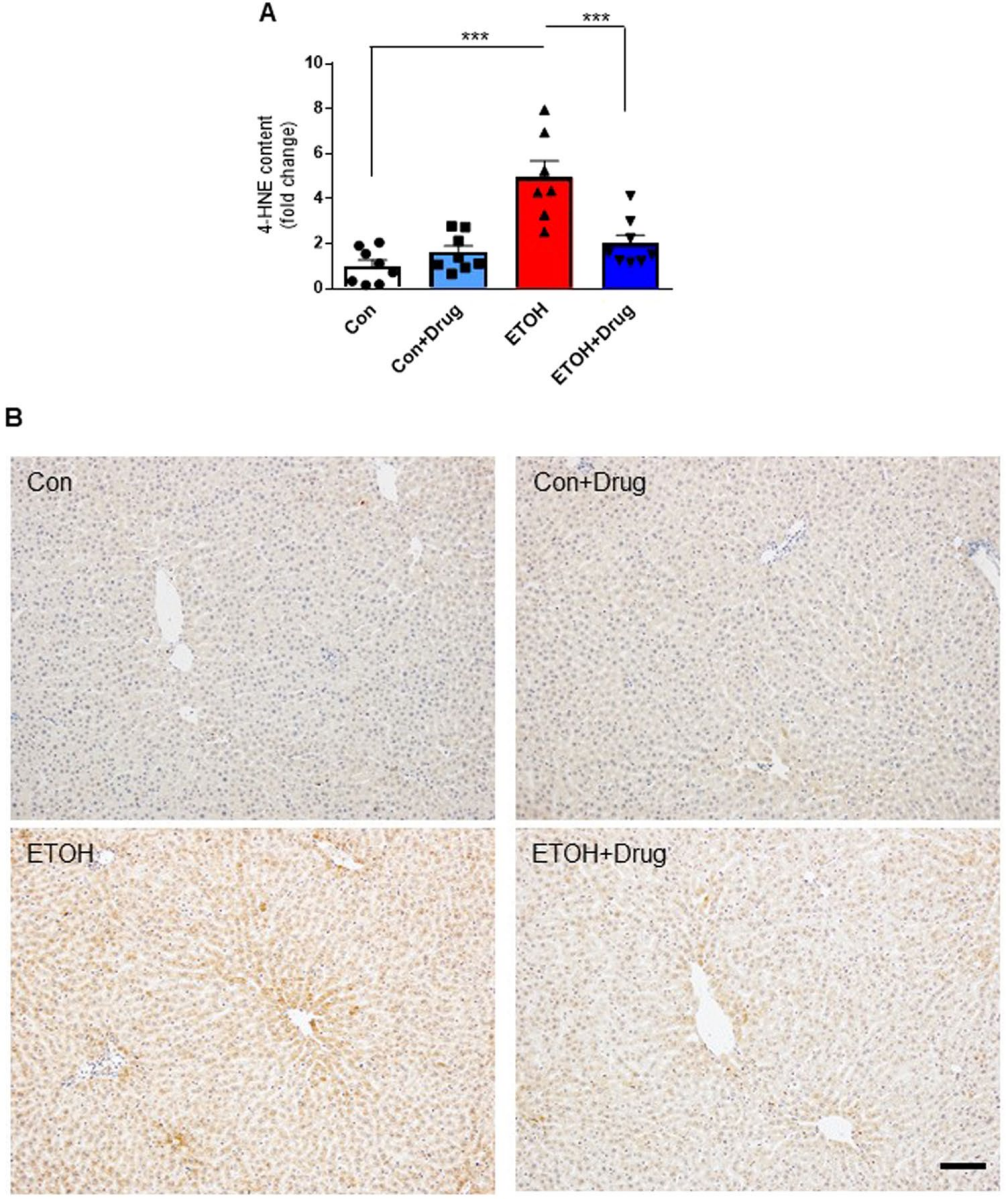
Effect of PCSK9 inhibitor on oxidative stress by PCSK9 inhibitor. (**A)** Quantification of 4-HNE content (*n* = 7 for ETOH and 8 for the other groups). Data are means ± S.E.M. ***P < 0.001. Statistical significance was determined by one-way ANOVA with Tukey’s posthoc test. **(B)** Representative images of 4-HNE stained sections from indicated groups. Scale, 50 µm.

**Figure 7:**
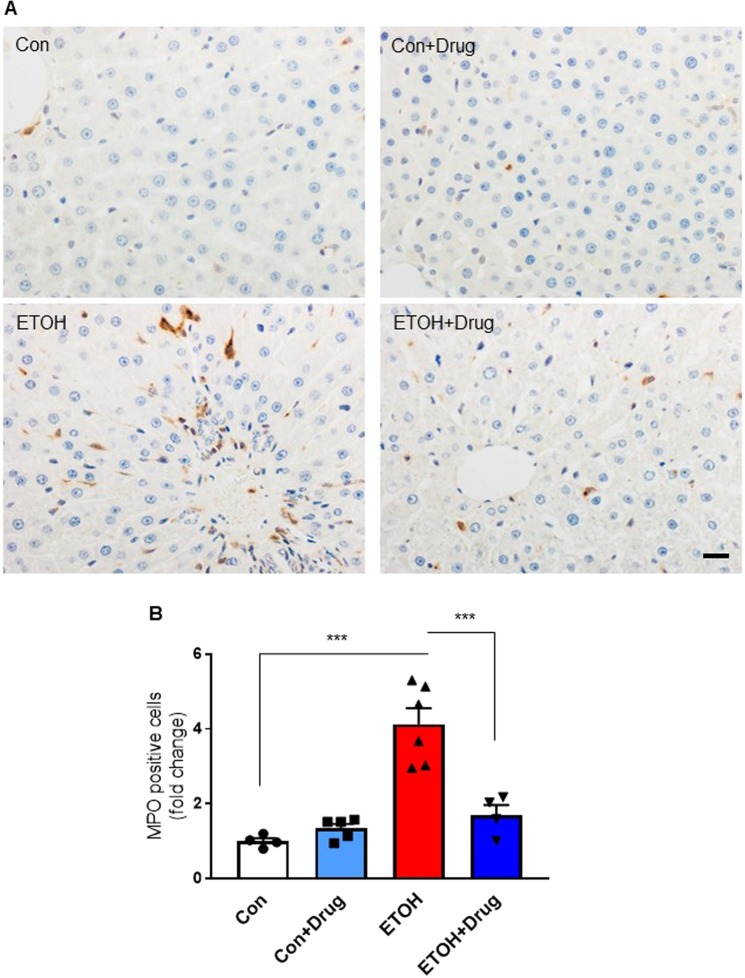
Effect of PCSK9 inhibitor on alcohol-induced neutrophil infiltration. (**A)** Representative immunostaining of MPO positive cells in liver sections from indicated groups. Scale bar, 20 µm. **(B)** Quantification of MPO positive cells expressed as fold change of the control group (*n* = 4 for Con and ETOH + Drug, 5 for Con + Drug, and 6 for ETOH). Data are means ± S.E.M. ***P < 0.001. Statistical significance was determined by one-way ANOVA with Tukey’s posthoc test.

Oxidative stress is one of the underlying mechanisms of alcohol-mediated liver damage^45,46^, and PCSK9 is closely involved in oxidative responses^47–49^. To study whether PCSK9 inhibition would influence oxidative stress caused by chronic alcohol consumption, we looked at 4-hydroxy-2-nonenal (4-HNE), a stable product of lipid peroxidation triggering cellular apoptosis in the liver. Alcohol feeding increased hepatic 4-HNE level and staining, which were attenuated by PCSK9 inhibitor treatment (Fig. 6A,B). These observations indicated attenuation of alcohol-induced hepatic oxidative stress by PCSK9 inhibition (Fig. 6A,B). We also measured MPO activity, a peroxidase enzyme present in neutrophils that generate reactive oxygen species^50^. The number of MPO positive cells was markedly higher in the alcohol-fed group than in the control diet-fed groups, whereas alirocumab treatment significantly reduced the number of infiltrating MPO positive cells (Fig. 7A,B).

### PCSK9 inhibition attenuates alcohol-induced liver inflammation

**Figure 8:**
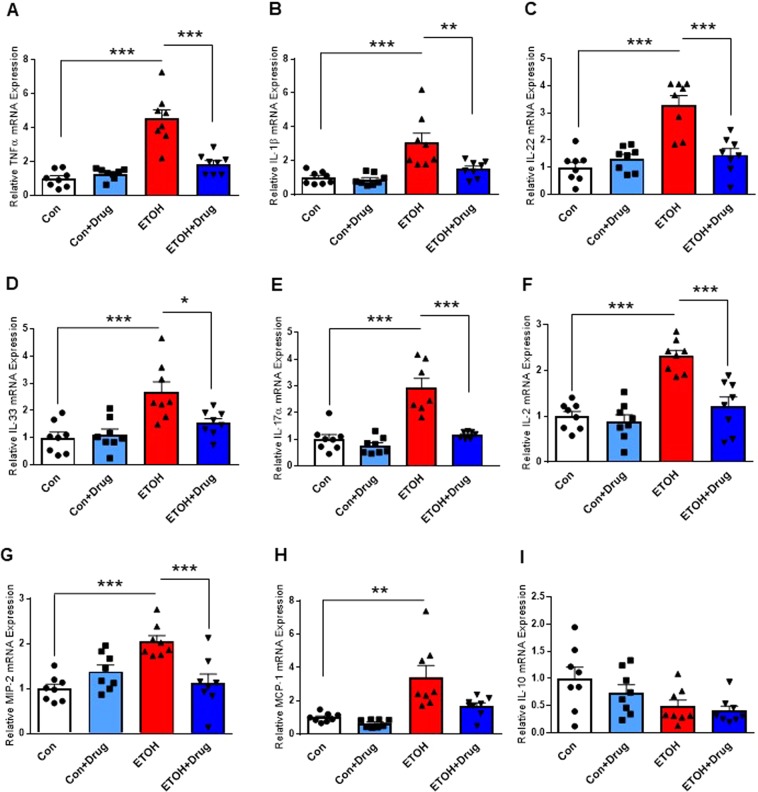
Effect of PCSK9 inhibitor on alcohol-induced liver inflammation. Hepatic mRNA levels of TNF-α **(A)**, IL-1β **(B)**, IL-22 **(C)**, IL-33 **(D)**, IL-17α **(E)**, IL-2 **(F)**, MIP-2 **(G)**, MCP-1 **(H)**, and IL-10 **(I)** (n = 8 for all groups except IL-17; IL-17, n = 7 for ETOH and ETOH + Drug and 8 for Con and Con + Drug). Data are means ± S.E.M. *P < 0.05, **P < 0.01, and ***P < 0.001. Statistical significance except MCP-1 and IL-10 was determined by one-way ANOVA with Tukey’s posthoc test. Statistical significance for MCP-1 and IL-10 was determined by Kruskal-Wallis test with Dunn’s posthoc test.

It has been reported that human PCSK9 promoted pro-inflammatory responses in cultured human macrophages by enhancing the expression of inflammatory cytokines and chemokines^51^. To identify whether PCSK9 would be involved in hepatic inflammatory responses by alcohol, we measured inflammatory markers in the liver. Chronic 12% alcohol feeding increased mRNA expression of proinflammatory cytokines (tumor necrosis factor alpha (TNF)-a, interleukin 1 beta (IL)-1β, IL-22, IL-33, IL-17α, IL-2), and chemokines (macrophage inflammatory protein (MIP)-2 and monocyte chemoattractant protein (MCP)-1), whereas alirocumab treatment attenuated alcohol-induced mRNA expression of the pro-inflammatory markers (Fig. 8A–H). Moreover, IL-10, an anti-inflammatory cytokine, decreased in the alcohol-fed group, but it did not reach the statistical significance (Fig. 8I). Consistently with the decreased number of hepatic MPO positive cells induced by alcohol consumption, alirocumab treatment also attenuated the alcohol-induced inflammation.

## Discussion

In the present study, we show that chronic alcohol exposure leads to increased PCSK9 expression in the liver using a 12% alcohol liquid diet rat model. We also demonstrate for the first time that treatment with the monoclonal antibody alirocumab significantly reduces hepatic PCSK9 expression and subsequently increases LDL-R expression in the liver, which is accompanied by attenuation of alcohol-induced liver steatosis, inflammation, oxidative stress, and hepatocellular injury.

PCSK9 has been primarily studied because of its important role in cardiovascular disease pathophysiology via regulation of LDL-C metabolism^22,52^. Lipid dysregulation and accumulation in liver are hallmarks of alcoholic liver injury^1^. In this study, we used the recent FDA-approved LDL-C lowering drug alirocumab to test its effects on alcohol-induced hepatocellular injury, lipid accumulation, inflammation, and oxidative stress. Compellingly, alirocumab decreased the alcohol-induced hepatic steatosis and the consequent metabolic gene dysregulation. The attenuating liver injury effects might be directly related to the mechanism of action of alirocumab, which blocks PCSK9 binding to the LDL-R and subsequently leads to upregulation of LDL-R and reduced LDL-C, thus improving lipid metabolism and reducing oxidative stress. Another effect might be the direct anti-inflammatory effects of anti-PCSK9 treatment. In fact, several reports have demonstrated a role of PCSK9 in inflammation, sepsis, immune function, and other aspects of lipoprotein metabolism^53–57^. Our data show upregulation of multiple inflammatory markers with alcohol exposure and significant reduction of inflammation following chronic treatment with alirocumab for 6 weeks. Although it remains unclear whether the protective effects of alirocumab on alcohol-induced liver injury are primarily related to LDL regulation, anti-inflammatory actions, or both, anti-PCSK9 treatment might represent a novel approach to target several domains of ALD and AUD.

Given that PCSK9 plays a pivotal role in cholesterol regulation and is a known driver of cardiovascular disease (CVD), it has been speculated that PCSK9 is implicated in non-alcoholic fatty liver disease (NAFLD) which shows similar pathogenesis and pathological spectra to ALD and an increased risk of CVD. A recent study reported that steatosis induced by high fat diet in mice increased hepatic and plasma PCSK9 expression, thus diminishing LDL-R expression and elevating plasma LDL-C^58^. Consistently with the preclinical finding, a positive correlation was observed between steatosis grade and circulating PCSK9 in subjects with a risk of non-alcoholic steatohepatitis (NASH)^57^. By contrast, Wargney *et al*. (2018) failed to demonstrate the significant association between circulating PCSK9 and hitological severity of NASH in metabolic high-risk populations^59^. The inconsistency between two studies may be accounted for differences in a degree of liver disases severity and a medication history of statin use between patient cohorts. Future studies should determine whether anti-PCSK9 treatment is also beneficial in NASH. Several reports have linked PCSK9 to extrahepatic functions, and it is plausible to postulate that anti-PCSK9 treatment might reduce alcohol-induced inflammation and tissue damage beyond the liver, including brain and other organs^53,54,60–62^. Future studies are needed to carefully evaluate these pleiotropic effects.

Despite its primary expression in liver^16^, the role of PCSK9 in liver disease is largely unknown. Zaid *et al*. (2008) indicated that PCSK9 was involved in liver regeneration^17^. In this study, PCSK9-deficient mice showed an impaired ability to regenerate liver tissue following partial hepatectomy and necrotic foci in the liver tissue. Notably, in prior studies the PCSK9 knockout mice were not properly backrossed to C57B/L6 background which is the optimal model for such studies. In contrast, in monkeys, alirocumab treatment in combination with statin did not worsen liver toxicity when compared to statin administration alone. Although liver sinusoidal cell hypertrophy was seen in rats between 2 and 5 weeks in the course of alirocumab administration, it disappeared by 3 months^32^. A possible explanation for the delay in the regeneration of liver tissue in the PCSK9 knockout mice may be attributed to low high-density lipoprotein cholesterol (HDL-C) because of the binding of apolipoprotein E containing HDL-C to the upregulated LDL-R^63^, which prevents liver from repairing properly after partial hepatectomy via regulation of precursors of liver sinusoidal cells^64^. Still, liver toxicity was not observed in monkeys despite markedly decreased HDL-C levels, and alirocumab administration did not reduce HDL-C in humans^32^. Therefore, the animal study using PCSK9-null mice (particully on an nonoptimal background for studies of liver inflammation and injury) may not be relevant clinically, and PCSK9 inhibitor could be considered as a safe pharmacological therapeutic for liver diseases.

Lipid metabolism is a complex process involving many pathways, and it has been long suggested that alcohol interferes with cholesterol homeostasis^65^. Our finding of upregulation of hepatic PCSK9 expression and downregulation of hepatic LDL-R after chronic alcohol exposure is consistent with previous reports. Wang *et al*. (2010) showed that rats exposed to a alcohol liquid diet for 4 weeks had alcohol-induced hypercholesteremia that was associated with reduced hepatic LDL-R and increased hepatic PCSK9 levels. Lohoff *et al*. (2018) showed in a chronic alcohol vapor model that hepatic PCSK9 was increased but in a NIAAA mouse model of chronic 10-day liquid diet^66^ and binge ethanol feeding that PCSK9 expression was decreased^15^. This discrepancy in hepatic PCSK9 levels could be attributed to distinct animal protocols of different duration specific for investigating different stage of ALD in 2 different species. Although blood alcohol levels in the vapored rats were maintained high, raning between 150 and 250 mg%^33^, plasma ALT and cytochrome P450 2E1 (CYP2E1, a marker for alcohol liver injury) showed a 2-fold increase in the alcohol-exposed group compared to the control group^67^. On the other hand, plasma ALT and AST were 4-fold elevated and hepatic CYP2E1 was remarkably upregulated in the group feeding chronic-plus-single-binge (binge alcohol-fed group) compared to a control group. Furtheremore, the binge alcohol-fed group exhibited markedly higher serum ALT and AST levels than the group chronically feeding alcohol only for 4 weeks^66^. Therefore, it is possible that the binge alcohol-fed mice in the NIAAA model experienced more severe alcohol damage than the alcohol-vapored rats, resulting in hepatic loss and alcohol liver toxicity observed in end-stage liver disease. In this study, we confirmed previous findings and showed that in the 12% alcohol liquid diet paradigm, PCSK9 and LDL-R were dysregulated.

Although it has been suggested that chronic alcohol exposure leads to increased activation of the transcription factor SREBP-2 and suppression of ERK1/2 activation^34^, the exact mechanism of alcohol-induced LDL-R dysregulation remains elusive. Of note, recent evidence also supports the hypothesis that LDL-R expression is regulated via PCSK9, which in turn is partially regulated through various transcription factors including SREBP-2 and hepatocyte nuclear factor-1α (HNF1α)^68–70^. We showed recently that individuals with AUD had abnormal methylation in the promoter region of PCSK9, precisely in the area where SREBP-2 and HNF1α bind, suggesting that alcohol might regulate PCSK9 expression via disruption of transcription factor binding sites. Moreover, it has been reported that other transcription factors, such as SREBP-1 and E2F1, are involved in PCSK9 expression in high-carbohydrate^35^ and in high-cholesterol diets^36^, repectively. Therefore, we tested the possible transcription factors for PCSK9 expression in the current study and found alirocumab reduced mRNA expression of alcohol-induced SREBP-1, SREBP-2, and E2F1, which suggests the potential signaling cascade involved in PCSK9 regulation.

To our knowledge, this is the first report testing anti-PCSK9 treatment in a model of alcohol-induced steatohepatitis and ALD. Interestingly, there have been some case reports of individuals with PCSK9 loss-of-function (LOF) mutations that were associated with liver steatosis in the context of obesity and type 2 diabetes^71,72^; however, individuals with LOF and hypocholesteremia do not exhibit excessive liver fat accumulation^73^. Some report show that triglyceride levels are reduced with low PCSK9 levels, whereas other reports demonstrate increase in hepatic triglyceride levels^17,57,74,75^. In our study, we did not see a significant reduction of triglycerides with just alirocumab treatment in the control diet-fed group; however, we observed an alcohol-induced increase in hepatic triglyceride content that was attenuated by alirocumab treatment. Furthermore, alirocumab admistration decreased alcohol-induced expression of the lipogenic enzyme (FAS) and restored downregulated β-oxidation modulators (PPARα and CPT1) by alcohol exposure. PCSK9’s role in alcoholic *versus* non-alcoholic steatohepatitis might differ and needs further exploration. Although alcohol exposure increased serum and hepatic lipids such as cholesterol, which was reduced by alirocumab administration, we could not find any statistically significant differences between groups. However, increased levels of hepatic LDL-R expression in the drug treated groups support the effectiveness of the PCSK9 inhibitor. We suspect overnight food consumption and nutritional status of the animals might affect these conflicting results. Indeed, serum and hepatic TG and cholesterol levels varied depending on fasting time^76^ and different composition of dietary fats^77^. Therefore, additional studies are needed to comprehensively assess plasma lipids in our model.

In summary, we show that anti-PCSK9 treatment via the monoclonal antibody alirocumab attenuates alcohol-induced hepatocellular triglyceride accumulation, inflammation, oxidative stress, and hepatocellular injury. Given the currently limited therapeutic options for individuals with ALD, despite recent advances in the understanding of the pathophysiology of ALD^78–80^, anti-PCSK9 treatment might provide a new therapeutic approach for the treatment of ALD. In particular, the use of a monoclonal antibody, which spares liver metabolism and has favorable side effect profile for liver adverse effects^81^, makes this a promising candidate for the treatment of alcohol-related liver disease. Future clinical studies in individuals with alcoholic and non-alcoholic liver disease are needed to evaluate efficacy and safety of anti-PCSK9 treatments in ALD/AUD.

## Supplementary information

supplementary information

## Data Availability

The datasets generated during and/or analysed during the current study are available from the corresponding author on reasonable request.
