# Supplementary material for: PCSK9 inhibition as a novel therapeutic target for alcoholic liver disease
Source: Sci Rep. 2019 Nov 20;9:17167. doi: 10.1038/s41598-019-53603-6 (PMC6868240; doi:10.1038/s41598-019-53603-6)

PCSK9 inhibition as novel therapeutic target for alcoholic liver disease

Ji Soo Lee, Partha Mukhopadhyay, Csaba Matyas, Eszter Trojnar, Janos Paloczi, Yuan Ru Yang, Brandon A. Blank, Cody Savage, Alexander V. Sorokin, Nehal N. Mehta, Janaina C.M. Vendruscolo, George F. Koob, Leandro F. Vendruscolo, Pal Pacher, Falk W. Lohoff

Supplementary Information. Full-length western blots

Supplementary Figure. Full-length blots

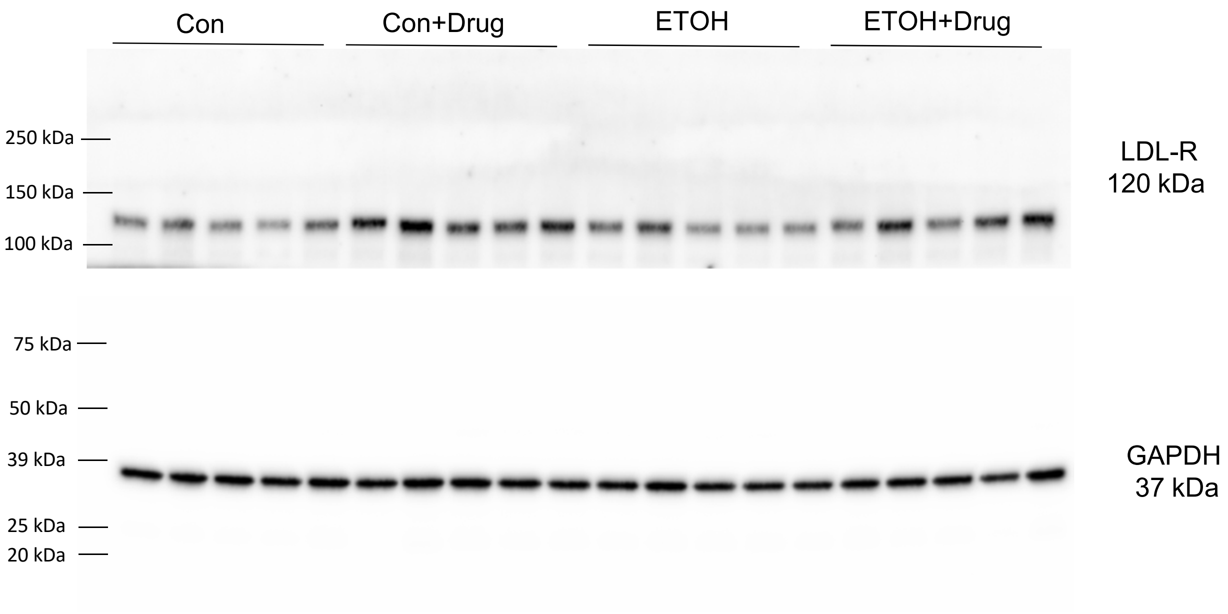

Supplement: Supplementary file 1 — supplementary information [file 41598_2019_53603_MOESM1_ESM.pdf]
